# Water Extractable Arabinoxylan Aerogels Prepared by Supercritical CO_2_ Drying

**DOI:** 10.3390/molecules18055531

**Published:** 2013-05-14

**Authors:** Jorge Marquez-Escalante, Elizabeth Carvajal-Millan, Mario Miki-Yoshida, Lorena Alvarez-Contreras, Alma Rosa Toledo-Guillén, Jaime Lizardi-Mendoza, Agustín Rascón-Chu

**Affiliations:** 1 Laboratorio de Biopolímeros, CTAOA, Centro de Investigación en Alimentación y Desarrollo, CIAD, A.C. Carretera a La Victoria Km. 0.6, Hermosillo, Sonora 83000, Mexico; E-Mails: jmarquez@estudiantes.ciad.mx (J.M.-E.); atoledo@ciad.mx (A.R.T.-G.); jalim@ciad.mx (J.L.-M.); 2 Centro de Investigación en Materiales Avanzados S.C. Miguel de Cervantes 120, Chihuahua, Chih., CP 31109, Mexico; E-Mails: mario.miki@cimav.edu.mx (M.M.-Y.); lorena.alvarez@cimav.edu.mx (L.A.-C.); 3 Laboratorio de Biotecnología, CTAOV, Centro de Investigación en Alimentación y Desarrollo, CIAD, A.C. Carretera a La Victoria Km. 0.6, Hermosillo, Sonora 83000, Mexico; E-Mail: arascon@ciad.mx

**Keywords:** ferulated arabinoxylans, supercritical drying, microstructure, texture, rehydration

## Abstract

Water extractable arabinoxylan (WEAX) aerogels were prepared by extracting the solvent from the alcogels (WEAX hydrogels with an alcohol as the solvent) with carbon dioxide under supercritical conditions. WEAX aerogels were characterized using scanning electron microscopy and adsorption and desorption nitrogen isotherms. The micrographs indicate a heterogeneous porous network structure in WEAX aerogel. Adsorption/desorption nitrogen isotherms of this material were type IV, which confirm that this material possess a mesoporous structure. WEAX aerogels rehydration capability was evaluated and the water absorption mechanism was determined. The WEAX aerogels water absorption mechanism was non-Fickian (n = 0.54).

## 1. Introduction

An aerogel is a highly porous solid material with exceptional surface area, suitable for loading active compounds [1]. Aerogels are produced by drying hydrogels, usually employing supercritical drying with CO_2_ (SC-CO_2_), which is able to avoid the structural collapse of material and maintains the porous texture of wet material [2,3]. All materials that can be obtained as hydrogels from solutions are potential candidates to form aerogels [4]. Aerogels can be produced from inorganic (e.g., inorganic resins, titanium dioxide, aluminum) or organic materials (e.g., carbon, polylactic acid, organic resins, polysaccharides) [3,5,6,7,8].

Among organic materials, natural polysaccharides have interesting properties such as stability, availability, renewability, low toxicity, biodegradability, biocompatibility and ability to be tailored as specific controlled delivery systems [9,10]. A polysaccharide with potential use as controlled delivery system and to our knowledge not yet explored as aerogel forming is arabinoxylan (AX). AX is constituted of a linear backbone of β-(1-4)-linked d-xylopyranosyl units to which α-l-arabinofuranosyl substituents are attached through O-3 and/or O-2,3 positions of the xylose residues [11]. Some of the arabinose residues are ester linked on (O)-5 to ferulic acid (FA) [12]. This polysaccharide has been classified as water extractable (WEAX) or water-unextractable (WUAX). One of the most important properties of WEAX is the ability to form hydrogels by covalent cross-linking involving FA oxidation by either chemical (ferric chloride, ammonium persulphate) or enzymatic (peroxidase/H_2_O_2_, laccase/O_2_) free radical-generating agents [11]. This oxidation allows the coupling of AX chains through the formation of dimers and trimers of FA (di-FA, tri-FA), generating an aqueous three-dimensional network. Furthermore there are physical interactions between AX chains that contribute to the stability of the network [13]. AX hydrogels are little affected by changes in temperature, ionic strength and pH [14]. In addition, AX hydrogels are neutral and have no odor or color [11]. Because of these characteristic and the macroporous structure of AX hydrogels, they have been proposed as matrices for controlled release of therapeutic proteins to be administered orally and further absorbed in the colon [13,14,15]. However, AX hydrogels have some disadvantages such as low stability in storage and low loading capacity [16,17]. An option that could eliminate such disadvantages is the formation of AX aerogels. This research is focused on the formation of WEAX aerogels by SC-CO_2_ and the study of their microstructure, textural properties and rehydration capability.

## 2. Results and Discussion

### 2.1. Extraction and Characterization of WEAX

Yield of WEAX extracted from wheat flour was 0.50% (w/w) on a dry matter basis (db, w WEAX/w wheat flour), which is in the range reported for other wheat WEAX [18]. WEAX composition is presented in Table 1. Pure arabinoxylan (AX) represented 67% db of the WEAX. The ratio arabinose-to-xylose (A/X = 0.67) and the ferulic acid (FA) content was similar to those previously reported for wheat endosperm WEAX [16,18]. The viscosimetric molecular weight (Mν) and intrinsic viscosity ([ɳ]) values were 748 kDa and 3.26 dL/g, respectively, which are in the range indicated for other wheat WEAX [11]. Small amounts of di-FA were detected (0.04 µg/mg WEAX) which is in agreement with earlier studies, suggesting that some arabinoxylan chains might be inter and/or intra cross-linked [16]. The relative percentages of each di-FA were: 69, 23 and 8% for the 8-5' (mainly in the benzofuran form), 8-O-4' and 5-5' structures, respectively. The 8-8' di-FA was not detected in this study. The predominance of 8-5' and 8-O-4' dimer structures has been previously reported in cereal arabinoxylans [13] Traces of a trimer of FA (tri-FA 4-O-8', 5'-5'') were registered (0.001 µg/mg WEAX). As presented in Table 1, the determined matter in WEAX was only 77% db, which is similar to that reported for other wheat flour WEAX [19].

**Table 1:** Composition of WEAX.

Component	Content
Arabinose ^a^	27.00 ± 1.10
Xylose ^a^	40.00 ± 0.40
Glucose ^a^	5.30 ± 0.30
Protein ^a^	4.70 ± 0.01
Ferulic acid ^b^	0.530 ± 0.001
Diferulic acids ^b^	0.040 ± 0.002

^a^ Results are expressed in g/100 g WEAX dry matter basis. ^b^ Phenolics are expressed in µg/mg WEAX dry matter basis.

### 2.2. WEAX Hydrogel

**Figure 1:**
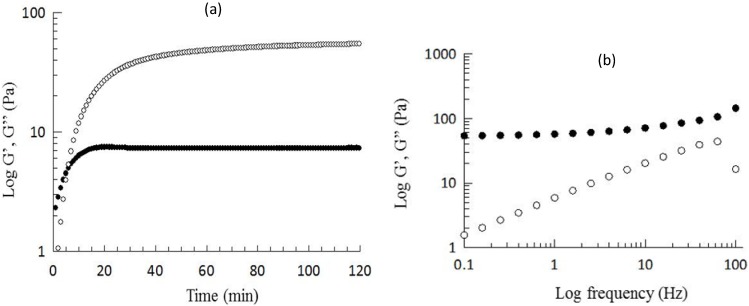
(**a**) Rheological (G'●, G''○) kinetics of 2% (w/v) WEAX solution gelation by laccase collected at 1Hz and (**b**) mechanical spectrum of the cured gel. Data obtained at 25 °C and 5% strain.

The kinetics of gelation of WEAX was monitored by small amplitude oscillatory shear rheology. Figure 1a shows the development of elastic (G') and viscous (G'') moduli of a 2% (w/v) WEAX solution undergoing gelation by laccase. WEAX gelation presented a characteristic kinetics with an initial increase of G' followed by a plateau region. The values of G' and G'' at the plateau region (120 min) were 55 and 7 Pa, respectively, which are in the range reported for other WEAX hydrogels [19]. The mechanical spectrum of the WEAX hydrogel (Figure 1b), was typical of a solid-like material with a linear G' independent of frequency and G'' much smaller than G' and dependent of frequency [20]. FA, di-FA and tri-FA were measured in WEAX hydrogels. FA oxidation by laccase generated di-FA (0.12 µg/mg WEAX) and traces of tri-FA (0.003 µg/mg WEAX) in the WEAX hydrogel. At the end of gelation, the 8-5' (principally benzofuran form), 8-O-4' and 5-5' dimers represented 76, 9 and 15% of the total amount of di-FA, respectively. The main increase in di-FA concerned the 8-5' form. The predominance of 8-5' dimers and absence of the 8-8' structure has been previously reported for WEAX gels [13,16]. The amounts of di-FA produced during gelation never counterbalanced the loss of FA. Indeed, at the end of gelation, 64% of the initial FA in the WEAX solution has disappeared, while only 37% was recovered as di-FA. Previous studies have also reported low ferulate recovery after oxidative treatment of WEAX [13,19,21]. These authors have suggested the formation of higher oligomers of ferulate.

### 2.3. WEAX Aerogel

**Figure 2:**
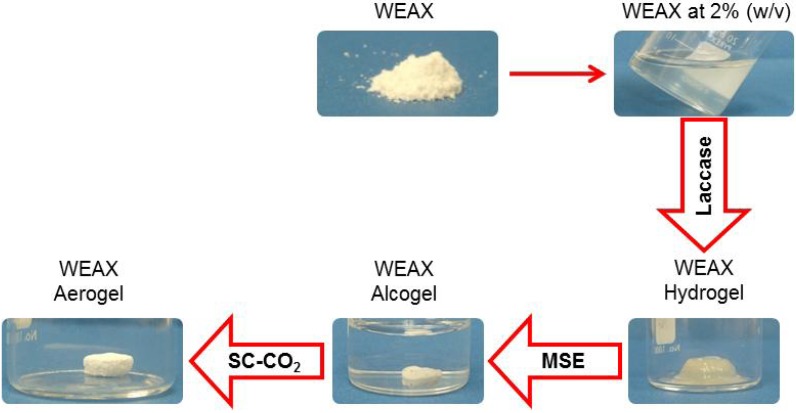
WEAX aerogel preparation.

WEAX aerogel preparation steps are presented in Figure 2. After WEAX hydrogel formation by laccase a WEAX alcogel was prepared by multistep solvent exchange (MSE) as reported elsewhere (Figure 2) [22]. WEAX aerogel was obtained by SC-CO_2_ drying of a WEAX alcogel by a modification of a previously reported method [23,24]. It has been reported that the SC-CO_2_ drying avoided the hydrogel structure to collapse, as occurs during hydrogels drying by other methods such as freeze drying [25]. On the other hand, ethanol was used instead of acetone during MSE because it has been reported that such solvent produce large gel volume reduction [26].

**Figure 3:**
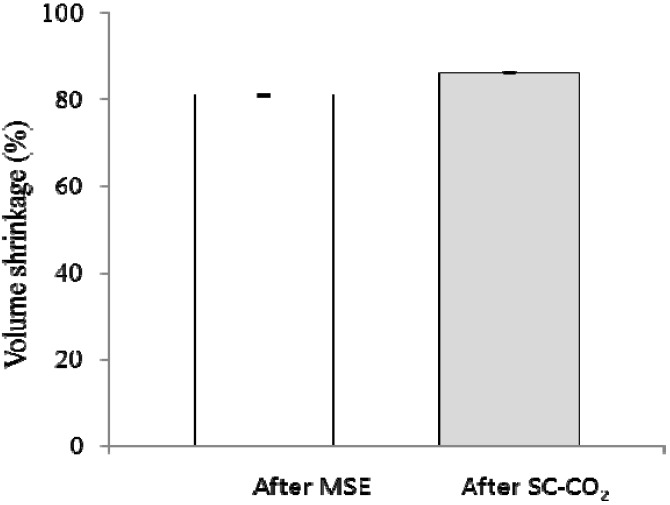
Volume shrinkage of WEAX hydrogel during drying process.

The percentage of volume shrinkage of WEAX hydrogels during drying process were 81 and 86% after MSE and SC-CO_2_ process, respectively (Figure 3). These values are higher than those reported for starch and alginate aerogels (77% and 64%, respectively) [22], but smaller than the value found for agar and carrageenan aerogels (95% and 92, respectively %) [27]. These authors suggest that aerogel volume shrinkage depends on the gel characteristics and the MSE process. For example, a high polysaccharide concentration in the gel could increase interaction between polysaccharide chains resulting in an increase of the network shrinkage.

### 2.4. Microscopy and Texture of WEAX Aerogel

**Figure 4:**
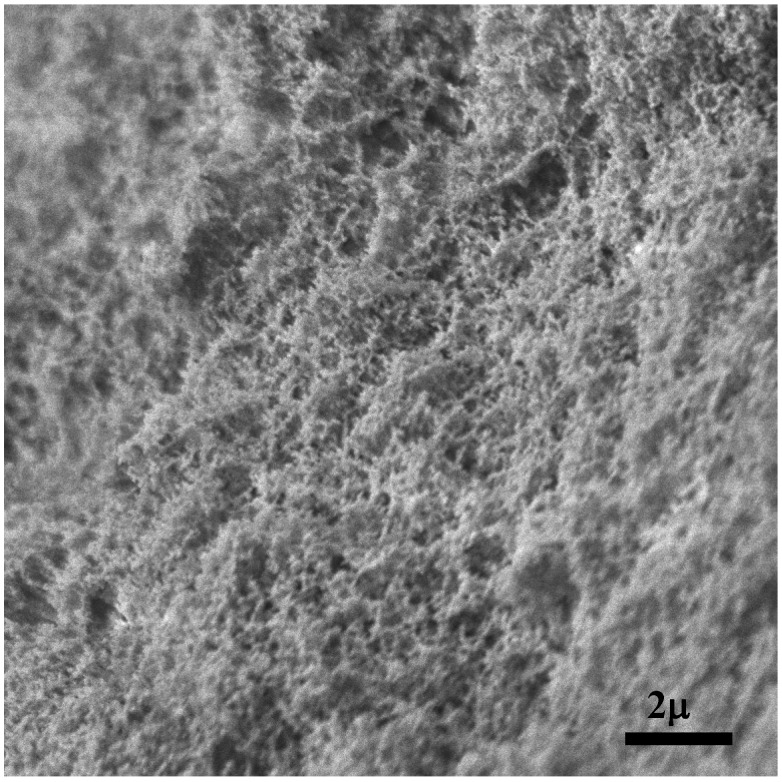
SEM image of WEAX aerogel.

Scanning electron microscopy (SEM) was applied to analyze the structure of WEAX aerogels (Figure 4). The secondary electron SEM micrograph shows the microscopic structure of these aerogels. Detailed examination of SEM micrographs suggests that the aerogels are constituted by the juxtaposition of globular nanoparticles, of around 110 nm, forming irregular chains of nanoparticles and consequently a porous structure. This porous network is different to that reported for lyophilized arabinoxylans hydrogels, which exhibit a honeycomb-like structure [28]. It has been suggested that the surface morphololgy of polysaccharide aerogels could depend on the preparation method [23,24].

**Figure 5:**
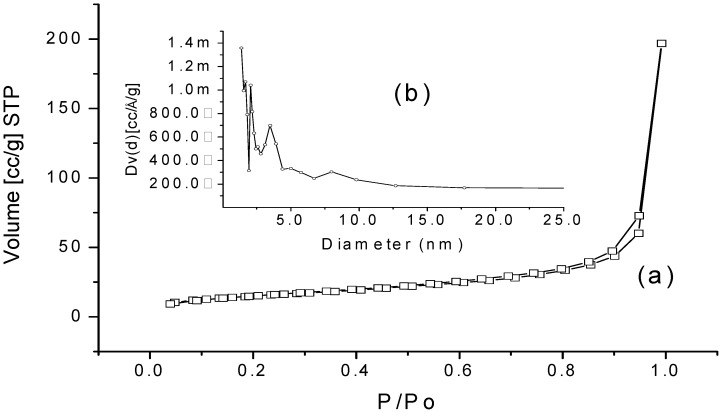
Textural analysis of WEAX aerogel. (**a**) Isotherm. (**b**) Pore size distribution.

WEAX aerogel presented a type IV adsorption/desorption isotherm (Figure 5a), which is a typical N_2_-isotherm reported for polysaccharide aerogels [27], indicating that this aerogel possess a mesoporous structure. Furthermore, the low developed hysteresis loop with a desorption step above the relative pressure of 0.7 shown in N_2_-isotherm is characteristic of a mesoporous organization [27,29]. The BJH pore size distribution for the WEAX aerogel confirms this observation (Figure 5b). The specific surface area value (*S_a_*), pore volume (*V_p_*) and average mesopore diameter (*P_d_*) of WEAX aerogel are presented in Table 2. As already reported, N_2_ physisorption isotherm provides information of the surface but does not provide full information on the structure of the surface. Generally, a better understanding of the texture of the aerogel can be obtained by combination with other characterization techniques. Moreover, the shape of the N_2_ physisorption isotherms provides information on the porosity of the system, although not all porosity can be detected by this technique. For instance, some cavities of the network of WEAX aerogel of Figure 4 are larger than 110 nm. Such cavities are too wide to be detected by N_2_ adsorption methods, which are limited to the analysis of mesopores with size between 2 and 50 nm [30]. The *V*_p_ value for WEAX aerogel was similar to those reported for pectin and starch aerogels (0.38 and 0.37 m^3^/g, respectively) [31]. WEAX aerogel *P_d_* mesopores value (2.2 nm) is close to starch aerogel value (1.9 nm) previously reported [22]. In contrast, WEAX aerogel *S*_a_ value was slightly lower than those reported in the literature for starch aerogel (90.3 m^2^/g) [22] and considerably lower than those reported for agar, pectin and alginate aerogels (320, 200 and 150 m^2^/g, respectively) [31]. In addition, other polysaccharides aerogels such as carrageenan, chitin, chitosan and glucan have been reported to show adsorption/desorption isotherms of nitrogen type IV [32,33,34]. In those studies higher values of *V_p_*, *P_d_* and *S_a_* were reported. The differences in *V_p_*, *P*_d_ and *S_a_* values among polysaccharide aerogels could be due to the MSE and drying process conditions, the polysaccharide characteristics and the gelling process.

**Table 2:** Textural properties of WEAX aerogel.

Textural property	Value
Pore volume, *V_p_* (cm^3^/g)	0.30
Average mesopore diameter, *P_d_* (nm)	2.20
Surface area, *S_a_* (m^2^/g)	53.70

### 2.5. Rehydration of WEAX Aerogel

**Figure 6:**
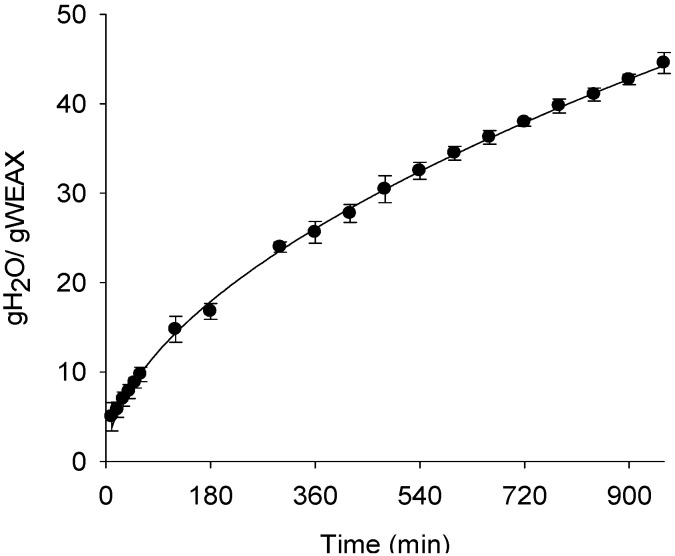
Rehydration and Fick model of WEAX aerogel in sodium azide at 0.02% (w/v). Water uptake (●); Fick model (--).

The rehydration of WEAX aerogels was followed as a function of time. During 15 hours WEAX aerogels registered a constant increase in water uptake (Figure 6). The n and k values of the Fick model for WEAX aerogel was calculated (Table 3). In contrast to n value for WEAX hydrogel (n = 0.37, data not shown), the n value for WEAX aerogel was superior to 0.45, indicating that the water uptake is due to a non-Fickian mechanism, where transport is dominated by diffusion-relaxation of polymeric chains. This result could be related to the presence of uncrosslinked polymer chains sections in the polysaccharide network [35].

**Table 3:** Fick model *n* and *k* values for rehydrated WEAX aerogel.

Parameter	Value
*k*	1.07 ± 0.10
*n*	0.54 ± 0.01
r^2^	0.99

## 3. Experimental

### 3.1. Materials

Water extractable arabinoxylans (WEAX) were obtained from Tacupeto wheat cultivar by using a previously reported procedure [19]. Laccase (benzendiol:oxygen oxydoreductase, EC 1.10.3.2) from *Trametes versicolor* and other chemical products were purchased from Sigma Chemical Co. (St. Louis, MO, USA).

### 3.2. Methods

#### 3.2.1. WEAX Characterization

Neutral sugar content in WEAX was determined by hydrolysis of the polysaccharides with 2 N trifluoroacetic acid at 120 °C for 2 h as reported before [19] and analyzed by high performance liquid chromatography (HPLC) using a Supelcogel Pb column (300 × 7.8 mm; Supelco, Inc., Bellefont, PA, USA) eluted with 5 mM H_2_SO_4_ at 0.6 mL/min and 50 °C. A Varian 9012 HPLC with Varian 9040 refractive index detector (Varian, St. Helens, Australia) and a Star Chromatography Workstation system control version 5.50 were used. Ferulic acid (FA), dimers of ferulic acid (di-FA) and trimers of ferulic acid (tri-FA) contents were determined in WEAX after saponification by RP-HPLC [13,16]. An Alltima C18 column (250 × 4.6 mm) (Alltech Associates, Inc., Deerfield, IL, USA) and a photodiode array detector Waters 996 (Millipore Co., Milford, MA, USA) were used. Detection was followed by UV absorbance at 320 nm. Protein content of WEAX was determined according to the Dumas method [36], using a NA 2000 nitrogen and protein analyzer (Fisons Instruments, Arcueil, France) (N × 5.7). Specific viscosity, *η*_sp_ was measured by registering the flow times of WEAX solutions in water (from 0.1 to 0.6% w/v) in an Ubbelohde capillary viscometer (OB size; Koehler Instrument, Bohemia, NY, USA) at 25 ± 0.1 °C. The intrinsic viscosity ([*η*]) was estimated from relative viscosity measurements, *η*_rel_, of WEAX solutions by extrapolation of Kraemer and Mead and Fouss curves to “zero” concentration. The viscosimetric molecular weight (*Mν*) was calculated from the Mark-Houwink relationship, *Mν* = ([*η*]*/k*)^1*/α*^ [19].

#### 3.2.2. WEAX Hydrogel

A WEAX solution at 2% (w/v) was prepared in 0.05 M citrate phosphate buffer pH 5. The formation of the WEAX gel was followed using a strain-controlled rheometer (Discovery HR-3 rheometer, TA instruments) in oscillatory mode as follows as reported before [13]. Cold (4 °C) solutions of 2% (w/v) WEAX were mixed with laccase (1.675 nkat per mg WEAX) and immediately placed in the cone and plate geometry (5.0 cm in diameter, 0.04 rad in cone angle) maintained at 4 °C. WEAX gelation kinetic was monitored at 25 °C for 2 h by following the storage (G') and loss (G'') modulus. All measurements were carried out at a frequency of 0.1 Hz and 5% strain (linearity range of viscoelastic behavior). Frequency sweep (0.16 to 16 Hz) was carried out at the end of the network formation at 5% strain and 25 °C. Reverse phase high-performance liquid chromatography (RP-HPLC) was used to quantify FA, di-FA and tri-FA contents in WEAX hydrogels after a deesterification step, as described elsewhere [13,16].

#### 3.2.3. WEAX Aerogel

After laccase addition, WEAX solutions were quickly transferred to a 1.5 mL tip-cut-off syringe (diameter 1.5 cm) and allowed to gel. After WEAX hydrogels were formed, WEAX alcogels were prepared by MSE as reported elsewhere [21]. MSE involved five steps: 30:70, 50:50, 70:30, 100:00 and 100:00 ethanol/water solutions. Exchange time of 3 hours was used, and only the last step was for overnight. The volume shrinkage of WEAX alcogels was measured by using a vernier caliper. WEAX alcogels were then dried by extraction with SC-CO_2_ according to previous reports [23,24]. Drying process was performed in a Dense Gas Management System (Marc Sims, Berkeley, CA, USA) by using the following conditions: 100 < p < 120 Bar, T = 40 ± 1 °C, a first flow rate step without CO_2_ during 4 h and a second flow rate step with CO_2_ at 4800 cc/min for 8 h. Finally, SC-CO_2_ was converted to gaseous form and later substituted by air when the system was opened at ambient conditions. The volume shrinkage of the WEAX aerogels was measured by using a vernier caliper.

#### 3.2.4. Microscopy and Textural Analysis of WEAX Aerogel

WEAX aerogels for scanning electron microscopy were disposed on aluminum stand employed conductive self-adhesive carbon label. Samples were examined without coating at low voltage (1.8 kV) in a JSM-7401F field emission scanning electron microscope (JEOL, Tokyo, Japan). SEM images were obtained in secondary electrons modes [28]. The textural properties measurements were conducted by adsorption/desorption of nitrogen. The specific surface area value (*S_a_*) of WEAX aerogel was determined by N_2_ adsorption using Brunauer-Emmett-Teller (BET) isotherm, while overall pore volume (***V_p_***) and average pore size (***P_d_***) were estimated by N_2_ adsorption using the Barrett-Joyner-Halenda (BJH) method. Surface area was determined by using nitrogen adsorption at their condensation temperature (77.35°K) and at a relative pressure (*p/p*_0_) of 0.5–0.22 [37]. The complete isotherm was conducted at *p/p*_0_ = 0.5–0.99 for adsorption and *p/p*_0_= 0.995–0.05 for desorption. A surface characterization Autosorb-1, Quantachrome was used. The surface of the samples was cleaned at 100 °C for 2 h under vacuum.

#### 3.2.5. WEAX Aerogels Rehydration

WEAX aerogels were allowed to swell in 20 mL of 0.02% (w/v) sodium azide solution to prevent microbial contamination. During 16 h the samples were blotted and weighted. After weighted, a new aliquot of sodium azide solution was added to the aerogels which were maintained at 25 °C during the test [19]. The water dissolution mechanism inward the WEAX aerogel was fitted to the following Fick model [38]:

Mt/Mo = *k*t*^n^*

where Mt is the weight of WEAX aerogel at time t, Mo is the initial weight of WEAX aerogel, *k* is the kinetic constant, and *n* is the dissolution exponent characteristic of the system. A value *n* < 0.45 indicates a Fickian dissolution mechanism, while 0.45 < *n* < 1 indicate a non-Fickian or anomalous mechanism.

#### 3.2.6. Statistical Analysis

Chemical determinations were made in triplicates and the coefficients of variation were lower than 7%. Small deformation measurements were made in triplicates and the coefficients of variation were lower than 9%. Rehydration tests were made in triplicates, coefficients of variation were lower than 10%. All results are expressed as mean values.

## 4. Conclusions

WEAX aerogels were prepared for the first time by supercritical CO_2_ drying of WEAX alcogels which were obtained from laccase induced WEAX hydrogels. WEAX aerogels present a porous structure constituted by the juxtaposition of nanoparticles of around 110 nm. These aerogels present a combined adsorption/desorption isotherms of nitrogen type II and IV indicating a heterogeneous porous organization. WEAX aerogels can be rehydrated through a non-Fickian mechanism.
